# The investigation of transcriptional repression mediated by ZEB2 in canine invasive micropapillary carcinoma in mammary gland

**DOI:** 10.1371/journal.pone.0209497

**Published:** 2019-01-15

**Authors:** Conrado de Oliveira Gamba, Karine Araújo Damasceno, Izabel Cristina Ferreira, Michele Angela Rodrigues, Dawidson Assis Gomes, Mariana Resende Alves, Rafael Malagoli Rocha, Alessandra Estrela Lima, Enio Ferreira, Geovanni Dantas Cassali

**Affiliations:** 1 Departamento de Patologia Geral, Instituto de Ciências Biológicas, Universidade Federal de Minas Gerais, Belo Horizonte, Minas Gerais, Brazil; 2 Curso de Medicina Veterinária, Universidade do Oeste de Santa Catarina, Campus aproximado de Campos Novos, Campos Novos, Santa Catarina, Brazil; 3 Laboratório de Patologia Experimental, Instituto Gonçalo Moniz, Fiocruz, Salvador, Bahia, Brazil; 4 Departamento de Bioquímica e Imunologia, Instituto de Ciências Biológicas, Universidade Federal de Minas Gerais, Belo Horizonte, Minas Gerais, Brazil; 5 International Center of Research in Cancer (CIPE), A.C. Camargo Cancer Center, São Paulo, São Paulo, Brazil; 6 Departamento de Patologia e Clínicas, Escola de Medicina Veterinária, Universidade Federal da Bahia, Salvador, Bahia, Brazil; Central University of Rajasthan, INDIA

## Abstract

The E-cadherin loss has frequently been associated with transcriptional repression mediated by transcription factors, such as the Zinc Finger E-Box Binding Homeobox-2 (ZEB2). Invasive micropapillary carcinomas (IMPCs) of the breast are aggressive neoplasms frequently related to lymph node metastasis and poor overall survival. In the canine mammary gland, IMPCs has just been reported and, based on its behavioral similarity with the human IMPCs, appears to be a good spontaneous model to this human entity. This study aimed to evaluate the relationship between E-cadherin and ZEB2 in a spontaneous canine model of invasive micropapillary carcinoma of the mammary gland. The correlation among gene expression (*ZEB2* and *CDH1)* and clinicopathological findings was also explored. Nineteen cases of IMPC of the canine mammary gland were obtained, protein and mRNA expression were investigated through immunohistochemistry and RNA *In Situ* Hybridization, respectively. To better understand the relationship between E-cadherin and ZEB2, immunofluorescence was performed in canine IMPCs. Immunohistochemically, most of IMPCs showed 1+ (14/19, 73.7%) for E-cadherin; and positivity for ZEB2 was diagnosed in 47.4% of the IMPCs. Regarding the RNA *In Situ* Hybridization (ISH), most of IMPCs showed 4+ and 0+ for E-cadherin (*CDH1*) and *ZEB2* respectively. Through immunofluorescence, the first and second more frequent combinatorial group were E-cadherin^+^ZEB2^-^ and E-cadherin^+^ZEB2^+^; neoplastic cells showing concomitantly weak expression for E-cadherin and positivity for ZEB2 were frequently observed. A negative correlation was observed between E-cadherin and progesterone receptor expression in IMPCs. Based on these results, canine mammary IMPCs show E-cadherin lost and, at times reveals nuclear positivity for the transcription factor ZEB2 that seems to exert transcriptional repression of the *CDH1*.

## Introduction

E-cadherin is a member of the cadherin transmembrane glycoproteins that mediate Ca^2+^-dependent cell-to-cell adhesion through homophilic interactions forming a major functional component of adherens junctions [1]. In woman´s breast cancer, this protein lost has been associated with unfavorable clinicopathological characteristics [2–5], poor overall survival [1–3,5–9], lymph node metastasis and distant metastasis [3,4,10–12]. The loss of E-cadherin has been associated with epigenetic mechanisms, such as the methylation of the *CDH1* (gene responsible for E-cadherin expression) promoters, changes in histones (methylation, acetylation, and ubiquitination), microRNA action and transcriptional repression mediated by a restricted group of transcription factors [13,14]. Included in this group are the molecules of the Zinc Finger E-Box Binding Homeobox family [Zinc Finger E-Box Binding Homeobox-1 (ZEB1) and Zinc Finger E-Box Binding Homeobox-2 (ZEB2)] that have as a target specific E-boxes, located in the proximal region of the promoter sequence of the *CDH1* [13,15,16].

The transcription factor ZEB2, besides the transcriptional repression in cancer, has been associated with malignant transformation of urothelial and ovarian human neoplasms; and with higher histological grade and advanced stage of colorectal carcinomas and gastric adenocarcinomas [17–20]. In breast cancer, a proclivity to poor overall survival has been reported in ZEB2-positive cases [21]. Interestingly, the *ZEB2* expression has also been related to lymph node metastasis in gastric, pancreatic and oropharyngeal neoplasms [17,22–24].

The Invasive micropapillary carcinomas (IMPC) of the breast are neoplasms rarely observed in the human species that are associated with high rates of lymphatic invasion (lymphotropism), lymph node metastasis and reduced overall survival [25,26]. In canine species, IMPC of the mammary gland has been reported showing biological behavior similar to its human counterparts [27–29]. To elucidate the mechanisms associated with the agressivity of human breast IMPC, canine mammary IMPC has recently been explored for our group revealing a decrease of E-cadherin expression and overexpression of EGFR and transcription factors (ZEB1, ZEB2 and SNAIL) [28,30,31]. The transcription factor SNAIL showed a relationship with E-cadherin downregulation and ZEB1 was associated with low histological grade [28,31]. Immunohistochemically, *ZEB2* cytoplasmic expression revealed association with poor overall survival. The *ZEB2* nuclear expression, important to transcriptional repression activity, has also been described [31], but its relationship with the *CDH1* downregulation should be better explored applying techniques that permit an investigation of the *ZEB2* and E-cadherin concomitantly; and that evaluate mRNA expression. Based on these findings, this study investigates the relationship between E-cadherin and ZEB2 in a spontaneous canine model of invasive micropapillary carcinoma of the mammary gland using mRNA *in situ* hybridization, immunohistochemistry and immunofluorescence. The correlation among gene expression (*ZEB2* and *CDH1)* and clinicopathological findings was also explored. This research demonstrated that IMPCs show E-cadherin lost and, at times reveals nuclear positivity for the transcription factor ZEB2 that seems to exert transcriptional repression of the *CDH1*.

## Materials and methods

### Case selection, clinical evaluation, and histopathological analysis

Nineteen cases of IMPC of the canine mammary gland were selected from the archives of the Veterinary School and the Laboratory of Comparative Pathology at the Institute of Biological Sciences of the Federal University of Minas Gerais and the Histopathology Laboratory of the Federal University of Bahia.

Histopathological analysis of IMPCs was performed as previously described with minor modifications [27,29]. The tumor specimens were previously fixed in 10% neutral buffered formalin and embedded in paraffin, and 4-μm-thick histological sections were cut and stained with hematoxylin and eosin. All IMPCs were reviewed and independently re-classified by two veterinary pathologists (COG and GDC) based on histopathological characteristics described by Misdorp [32] and Cassali et al. [33]. In brief, carcinomas presenting cystic formations containing nests of epithelial cells that reveal moruliform appearance (infiltrating micropapillary pattern), associated or not with ‘in situ’ micropapillary areas, were diagnosed as IMPCs. Pure (carcinomas with ≥ 75% infiltrating micropapillary pattern) and ‘mixed’ (carcinomas with < 75% infiltrating micropapillary pattern, associated with other infiltrating carcinomas) subtypes of IMPC were included in this study [27]. Canine IMPCs associated with clinical signs of inflammatory carcinoma were excluded.

### Immunohistochemistry

Immunohistochemistry was performed as previously described with minor modifications [28,29,31]. Sections (4 μm) of primary tumors were mounted on silanized slides and a peroxidase-based detection system, Advance HRP, was applied (Dako, Carpinteria, California, USA). The slides were dewaxed in xylene, and endogenous peroxidase activity was blocked with H_2_O_2_ 3% in methanol. The reagents were applied manually, and immunoreactivity was visualized by incubating the slides with 3,3’-diaminobenzidine (Lab Vision DAB substrate system; Lab Vision, Fremont, California, USA) for 10 min. The antibodies used were mouse monoclonal anti-E-cadherin (clone 4A2C7, Invitrogen, 1:80) and rabbit polyclonal anti-ZEB2 (Sigma–Aldrich, 1:100). Regarding antigen retrieval, for E-cadherin and ZEB2 the samples were incubated in a water bath (98°C) for 1 and 3 h, respectively. For E-cadherin, canine normal mammary gland was used as an internal positive control. Sections from ZEB2-positive canine tissues were used as external positive controls. Negative controls were performed using a normal serum (Lab Vision Ultra V Block) in place of the primary antibody.

For both markers, the immunohistochemical analysis was performed only in invasive areas. E-cadherin expression was classified based on the percentage of epithelial cells showing immunoreactivity of the cell membrane. Immunolabeled slides were scored as negative, having no detectable labeling; +1, detectable labeling in ≤ 10% of the neoplastic cells; +2, detectable labeling in 10–50% of the neoplastic cells; or +3, detectable labeling in > 50% of the tumor cells [34]. For ZEB2, positivity was assessed based on the presence (+) or absence (-) of labeling [21,35].

### Confocal microscopy

Confocal microscopy was performed as previously described with minor modifications [30]. In Brief, FFPE tissue sections were dewaxed, rehydrated and unmasked in Trilogy solution (Cell Marque, Koclin, CA, USA) in pressurized heating (125°C) during 20 min according to manufacturer’s instructions. Next, samples were rinsed in Phosphate Buffered Saline (PBS) (Sigma-Aldrich, Carlsbad, CA, USA), and then incubated in PBS containing 0.2% Triton X-100 (Sigma-Aldrich) for another 20 min and blocked in PBS containing 1% Bovine Serum Albumin (BSA, Sigma-Aldrich) for 30 min. The sections were next incubated with a mouse monoclonal antibody against E-cadherin (1:80, clone 4A2C7, Invitrogen) and a rabbit polyclonal antibody against ZEB2 (1:200, polyclonal, Sigma-Aldrich), overnight at 4°C, and then rinsed 3 times for 5 min in PBS. Subsequently, sections were incubated with Alexa Fluor 488 Goat Anti-mouse IgG antibody (1:1000, Life Technologies), Alexa Fluor 555 Goat anti-rabbit IgG antibody (1:1000, Life Technologies) and, Hoechst 33258 (1 μg/mL, Life Technologies) for 1 h at room temperature. Next, samples were washed 3 times in PBS for 10 min and then mounted in Prolong Gold Antifade reagent (Life Technologies). The negative control was included in all reactions, by omitting primary antibodies. Images were collected using a Zeiss LSM 5 Live (Carl Zeiss, Jena, Germany) confocal microscope using an oil 40x 1.3 NA objective lens. Samples were excited at: 405 nm and observed at 415–480 nm to detect Hoechst, 488 nm and observed at 500–525 nm to detect Alexa Fluor 488 and, at 532 nm and observed using an LP 550 filter to detect Alexa Fluor 555. For ZEB2 and E-cadherin, nuclear and cytoplasmic membrane staining was considered, respectively. Neoplastic epithelial cells were classified into four combinatorial phenotypic groups: E-cadherin^+^/ZEB2^+^, E-cadherin^+^/ZEB2^-^, E-cadherin^-^/ ZEB2^+^ and E-cadherin^-^/ ZEB2^-^. The number of neoplastic epithelial cells belonging to each group was counted in 10 invasive areas of IMPC using the Image J software (NIH, Baltimore, MD). Aiming to elucidate the role of ZEB2 in the loss of E-cadherin we evaluated the number of neoplastic cells showing concurrently nuclear ZEB2 and weak E-cadherin expression (ZEB2^+^/E-cadherin^weak^); and, nuclear ZEB2 and strong E-cadherin expression (ZEB2^+^/E-cadherin^strong^). The E-cadherin expression was considered strong when showed an intensity similar to those observed in the normal mammary gland.

### RNA *in situ* hybridization

RNA *In Situ* Hybridization method (ISH) was performed as previously described with minor modifications [36,37]. The RNAscope (Advanced Cell Diagnostics, Inc., Hayward, California) approach was used in archival formalin-fixed, paraffin-embedded (FFPE) tissue to view ZEB2 and E-cadherin mRNA in individual cells through a probe design strategy and hybridization-based on a signal amplification system to amplify signals and suppress background (ZEB2: the reference sequence, XM_005631964.1; probe region, 1362–2409; *CDH1*: the reference sequence, NM_001287125.1; probe region, 1347–2279). FFPE tissues sections four micrometers thick were deparaffinized in xylene, followed by dehydration in an ethanol series. Afterwards, tissue sections were incubated in a pre-treatment buffer maintained at a boiling temperature (100°C to 104°C) using a hot plate for 15 min, rinsed in deionized water, and immediately treated with a solution of pre-treatment 3, which consists of a protease enzyme at 40°C for 30 min in a HybEZ hybridization oven (Advanced Cell Diagnostics, Inc., Hayward, California). Thus, the tissue was able to be incubated with the target probes that lasted for 2 h at 40°C in a HybEZ hybridization oven (Advanced Cell Diagnostics, Inc., Hayward, California). After each hybridization step, slides were washed with wash buffer two times at room temperature. Preamplifier and amplifier molecules were hybridized in each probe pairs. Chromogenic detection was performed using diaminobenzidine (DAB), followed by counterstaining with Gill´s hematoxylin. Assays using archival FFPE specimens were typically performed in parallel with positive and negative controls to ensure interpretable results. The endogenous housekeeping gene was used as a positive control to assess both tissue RNA integrity and assay procedure, and the negative control was used to assess background signals. Staining results were evaluated, in invasive areas of IMPCs, by examining tissue sections under a standard bright field microscope at 20–60X magnification and categorized into five scores: (0) No staining or less than 1 dot to every 10 cells (60X magnification), (1+) 1–3 dots/cell (visible at 20–60X magnification), (2+) 4–10 dots/cell and very few dot clusters (visible at 20–60X magnification), (3+) >10 dots/cell and less than 10% positive cells have dot clusters (visible at 20X magnification) and (4+) >10 dots/cell and more than 10% positive cells have dot clusters (visible at 20X magnification). The evaluation method was adapted from the manufacturer's guideline.

### Statistical analysis

The GraphPad Prism 5.0 software package was used for the statistical analysis. A comparison between E-cadherin^+^/ZEB2^+^, E-cadherin^+^/ZEB2^-^, E-cadherin^-^/ZEB2^+,^ and E-cadherin^-^/ZEB2^-^ groups were performed using a two-sided non-parametric Kruskal-Wallis test. To compare E-cadherin^strong^/ZEB2^+^ and E-cadherin^weak^/ZEB2^+,^ the non-parametric Mann Whitney test^,^ was used. Spearman's correlation test was applied to estimate the relationship between clinicopathological findings, immunohistochemistry and ISH results for E-cadherin and ZEB2. Probability values below 0.05 were considered significant for all statistical tests.

### Ethical aspects

All procedures were performed under the guidelines and with the approval of the Ethics Committee in Animal Experimentation of the Federal University of Minas Gerais (CETEA/UFMG), protocol 0050/11.

## Results

### Epidemiological, clinicopathological and immunohistochemical findings of IMPCs

Some epidemiological and clinicopathological data were not available for all cases obtained. Age of animals ranged from 8–14 years old (mean 11 ± 1.9) at the time of surgery, and most were purebred female dogs (16/18, 89%). The average size of IMPCs was 5.8 ± 3.2 cm. Regional and distant metastases were observed in 100% (14/14) and 22% (4/10) of the dogs, respectively. Regarding the histopathological analysis of IMPCs, histological grade II (12/19, 63%) and the pure subtype (14/19, 74%) were more frequently observed. Thirteen IMPCs (13/13, 100%) were positive for Estrogen Receptor (ER) and Progesterone Receptor (PR). In six cases it was not possible to evaluate *ER* and *PR* expression. Regarding *c-erb-B2*/*HER-2* expression 10/14 (71%), 3/14 (21%) and 1/14 (7%) cases were 1+, 2+ and 3+, respectively. In five cases it was not possible to evaluate *c-erb-B2*/*HER-2* expression. The median overall survival of the canines was 135 days, and 13 animals died due to the disease; one animal was alive at 196 days after surgery, and one animal died due to a hemorrhagic diathesis. More details of the clinicopathological results of IMPCs are presented in Table 1.

**Table 1:** Clinicopathological features of canine mammary IMPCs.

Case	Breed	Age (years)	Lymph node metastasis	Tumour size (cm)	Distant Metastasis	Subtype^a^	Histological Grade^[^^38^^],[^^39^^]^	ER (Score/%)^b^^[^^40^^]^	PR (Score/%)^b^^[^^40^^]^	c-erb-B2/HER-2 (Score)^c^^[^^41^^]^	Survival(days)^d^
**1**	NA	13	NA	NA	NA	Pure	I	+ (90)	+(60)	1+	30
**2**	Labrador Retriever	12	Yes	7.5	NA	Pure	II	+(70)	+(30)	1+	60
**3**	Cocker Spaniel	13	Yes	7.3	NA	Pure	II	+(1)	+(90)	2+	NA
**4**	Pinscher	13	NA	4.5	NA	Pure	II	NA	NA	NA	NA
**5**	Poodle	12	Yes	2.5	NA	Pure	I	NA	NA	NA	330
**6**	Akita	8	NA	4	No	‘Mixed'	II	+(40)	+(90)	1+	8
**7**	Bichon Frise	10	NA	2	NA	Pure	II	+(80)	+(90)	1+	90
**8**	Cocker Spaniel	13	Yes	10	No	Pure	II	+(60)	+(60)	2+	180
**9**	Crossbreed	11	Yes	3	NA	Pure	III	+(60)	+(80)	1+	120
**10**	Siberian Husky	13	Yes	4	NA	Pure	II	NA	NA	NA	30
**11**	Dobermann	10	Yes	NA	Yes	Pure	III	(+)50	+(50)	1+	NA
**12**	Dalmatian	9	Yes	13	NA	‘Mixed'	II	+(1)	+(70)	2+	NA
**13**	Poodle	9	Yes	NA	Yes	‘Mixed'	I	NA	NA	NA	30
**14**	Poodle	13	Yes	6	Yes	Pure	III	+(90)	+(80)	1+	404
**15**	Poodle	12	Yes	3	Yes	‘Mixed'	II	+(60)	+(50)	1+	188
**16**	Dachshund	8	Yes	4	No	‘Mixed'	II	NA	NA	1+	150
**17**	Poodle	14	Yes	5	No	Pure	II	+(60)	+(40)	3+	71
**18**	Crossbreed	11	Yes	11	No	Pure	II	NA	NA	NA	360
**19**	Boxer	9	NA	6	No	Pure	I	+(20)	+(30)	1+	196

NA, Not Available; ER, Estrogen Receptor; PR, Progesterone Receptor

^a^ Pure subtype: carcinomas with a ≥75% infiltrating micropapillary pattern; ‘mixed’ subtype: carcinomas with a <75% infiltrating micropapillary pattern, associated with other infiltrating carcinomas

^b^ Cases were scored positives if nuclear staining was present in ≥1% of the tumor cells

^c^ HER-2 expression were determined by cell membrane staining and scored according to the guidelines established by the American Society of Clinical Oncology, College of American Pathologists (ASCO/CAP)

^d^ The 1,2,5,7,8,9,10,13,14,15,16,17,18 canines died due to the disease. The case 6 died due to another cause, and the canine 19 is alive.

### Immunohistochemistry expression analysis for ZEB2 and E-cadherin in invasive areas of the primary IMPCs of the canine mammary glands

**Fig 1:**
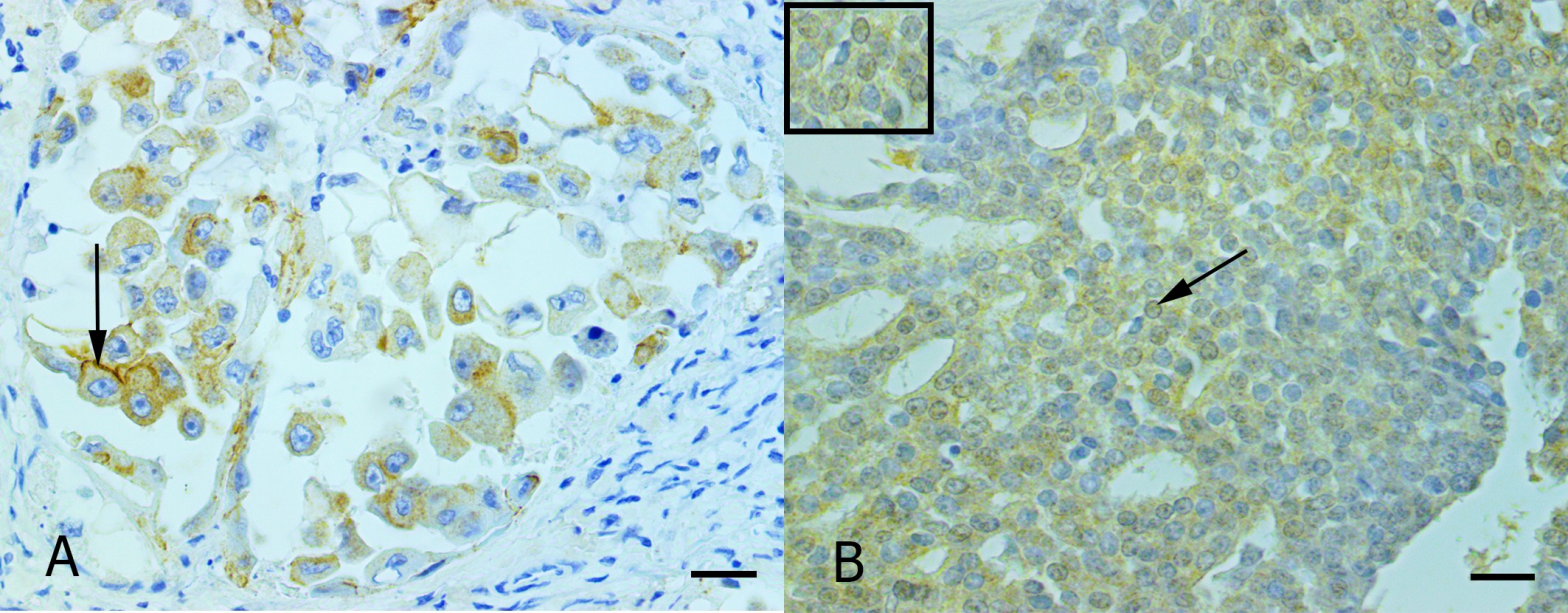
Photomicrographs illustrating E-cadherin and ZEB2 immunostaining of micropapillary carcinomas of the canine mammary gland. (A) Invasive micropapillary areas characterized by a decrease of E-cadherin expression. Some cells show high-intensity cytoplasmic membrane E-cadherin expression (arrow). This case was classified as 1+. Advance HRP peroxidase system and anti-E-cadherin; counterstained with Harris’s hematoxylin; scale bar, 30 μm. (B) The invasive micropapillary area is exhibiting neoplastic cells with the nuclear expression for ZEB2 (arrow). The inset shows details of the staining. Advance HRP peroxidase system and anti-ZEB2; counterstained with Harris’s hematoxylin; scale bar, 30 μm.

Concerning immunohistochemical evaluation, most of IMPCs were 1+ (14/19, 73.7%) for E-cadherin (Fig 1A), while 0%, 10.5% (2/19) and 15.8% (3/19) were 0+, 2+ and 3+, respectively. In total 47.4% (9/19) of IMPCs showed nuclear positivity for ZEB2 (Fig 1B). Interestingly fifty percent (7/14) of the E-cadherin 1+ IMPCs were ZEB2-positives. Statistical correlation between the protein expression of E-cadherin and ZEB2 was not observed (p = 0.68; r = - 0.10).

### Immunofluorescence analysis for ZEB2 and E-cadherin in invasive areas of the primary IMPCs of the canine mammary glands

**Fig 2:**
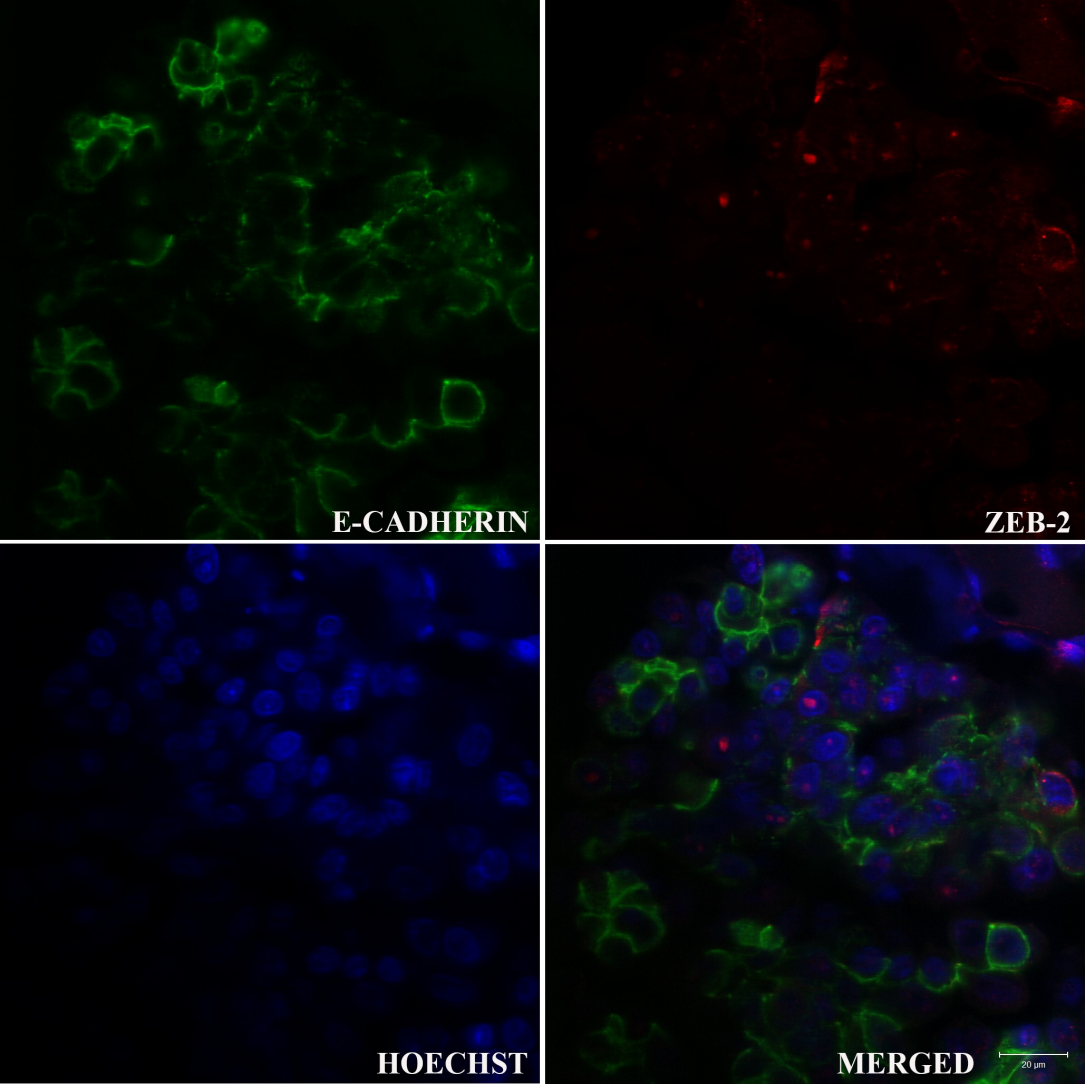
ZEB2 and E-cadherin expression in IMPC of the canine mammary gland. Confocal immunofluorescence image of the cytoplasmic membrane E-cadherin (green) (A), nuclear ZEB2 (red) (B) and nuclear staining with Hoechst (blue) (C). Note that most of the neoplastic cells present both, positivity for ZEB2 and negativity for E-cadherin. Some neoplastic cells reveal concomitantly positivity for ZEB2 and weak intensity E-cadherin expression. Nuclear localization of ZEB2 was confirmed for the merged image (D) that demonstrates the co-localisation of ZEB2 with Hoechst. Images are representative of what was observed in 10 fields of an IMPC case. Scale Bar = 20 μm.

To evaluate the relationship between E-cadherin downregulation and the transcriptional repression of ZEB2, a triple staining immunofluorescence analysis was performed in the 19 cases of IMPCs. Most of IMPCs showed a predominance of E-cadherin^+^ZEB2^-^ (16/19, 84%) neoplastic epithelial cells (p<0.0001); E-cadherin^+^ZEB2^+^ was the second more frequent combinatorial group (10/19, 53%) (Fig 2) (Table 2). Based on these results, to elucidate the role of ZEB2 in loss of E-cadherin expression, we dichotomized E-cadherin^+^ZEB2^+^ group, and most of the canine IMPCs showed a predominance of nuclear ZEB2^+^ E-cadherin^Weak^ (12/16, 75%) (p = 0.10) neoplastic cells (Table 3).

**Table 2:** Number of neoplastic epithelial cells stained for E-cadherin (ECAD) and ZEB2 in immunofluorescence analysis performed in invasive areas of IMPC of the canine mammary gland.

Case	Number of neoplastic epithelial cells counted*				
	Total	ECAD^-^ZEB2^-^	ECAD^-^ZEB2^+^	ECAD^+^ZEB2^-^	ECAD^+^ZEB2^+^
**1**	551	21	3	490	37
**2**	941	24	2	801	114
**3**	430	46	14	340	30
**4**	309	15	3	211	80
**5**	228	11	6	132	79
**6**	715	86	61	208	360
**7**	1001	365	0	636	0
**8**	624	53	9	418	144
**9**	238	39	0	194	5
**10**	755	133	104	404	114
**11**	381	50	33	117	181
**12**	1510	45	15	1382	68
**13**	328	111	14	172	31
**14**	609	254	0	355	0
**15**	368	90	66	116	96
**16**	546	511	0	35	0
**17**	817	62	10	580	165
**18**	305	18	19	153	115
**19**	413	138	17	239	19

*Total number of neoplastic epithelial cells counted in 10 fields of invasive areas of IMPC of the canine mammary glands.

**Table 3:** Number of neoplastic epithelial cells stained for E-cadherin (ECAD) and ZEB2 concomitantly (ECAD^+^ZEB2^+^) and their distribution in ECAD^Strong^ZEB2^+^ and ECAD^Weak^ZEB2^+^ groups based on the staining intensity for E-cadherin through immunofluorescence in invasive areas of IMPC of the canine mammary gland.

Case^#^	Number of neoplastic epithelial cells counted*		
	ECAD^+^ZEB2^+^	ECAD^Strong^ZEB2^+^	ECAD ^Weak^ZEB2^+^
**1**	37	5	32
**2**	114	68	58
**3**	30	6	24
**4**	80	34	46
**5**	79	48	31
**6**	360	82	278
**8**	144	58	86
**9**	5	3	2
**10**	114	21	93
**11**	175	51	124
**12**	68	18	50
**13**	31	14	17
**15**	96	45	51
**17**	165	35	130
**18**	115	61	54
**19**	19	0	19

NA, Not Available.

^#^The cases 7, 14 and 16 were not added because did not show ECAD+ZEB2+ cells

*Total number of neoplastic epithelial cells counted in 10 fields of invasive areas of IMPC of the canine mammary glands.

### mRNA *in situ* hybridization expression analysis for *ZEB2* and *CDH1* in invasive areas of the primary IMPCs of the canine mammary glands

**Fig 3:**
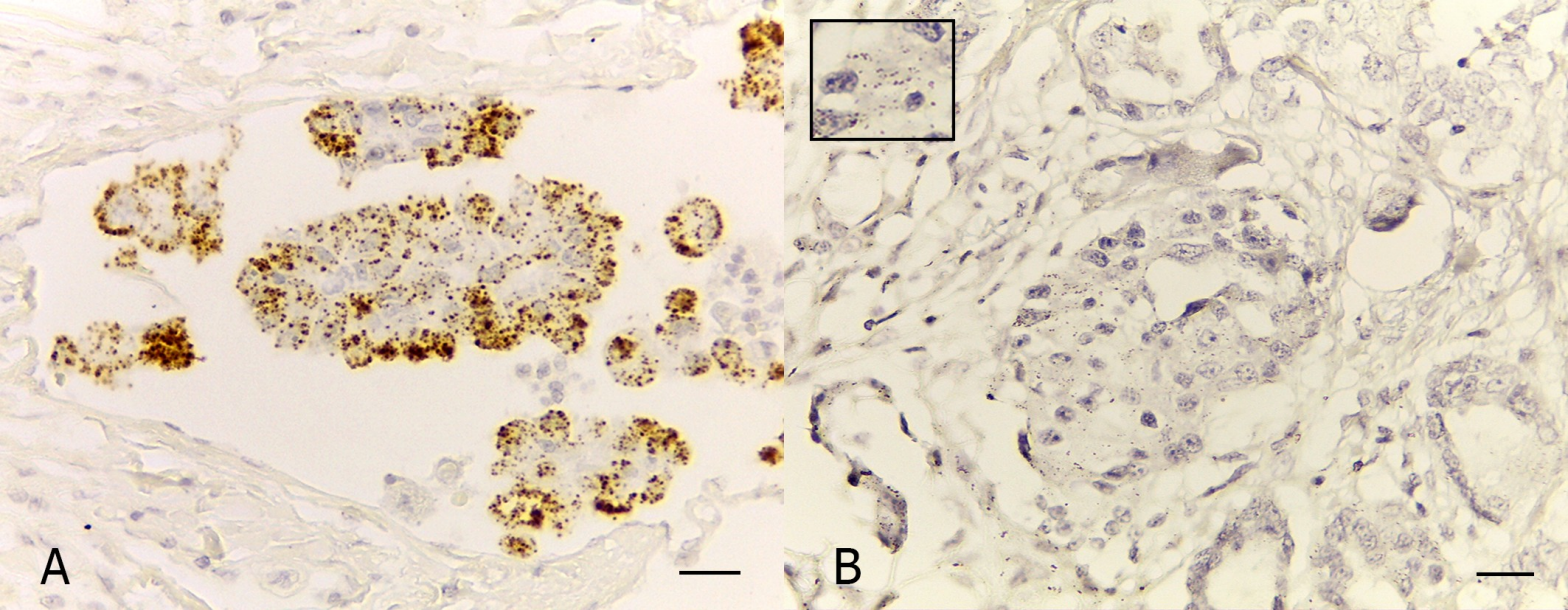
Photomicrographs illustrating *CDH1* and *ZEB2* mRNA expressions in micropapillary carcinomas of the canine mammary gland. (A) Invasive micropapillary area of a IMPC classified as 4+ for E-cadherin. The case reveals >10 dots/cell and more than 10% positive cells with dot clusters. RNAscope (Advanced Cell Diagnostics) approach; counterstained with Gill´s hematoxylin; scale bar, 30 μm. (B) Invasive micropapillary area of a IMPC classified as 2+ for *ZEB2*. The neoplastic cells show 4–10 dots/cell and very few dot clusters. The inset shows details of the staining. RNAscope (Advanced Cell Diagnostics) approach; counterstained with Gill´s hematoxylin; scale bar, 30 μm.

The RNA *In Situ* Hybridization method was applied in 15/19 (78.9%) cases of IMPC. As concerns *CDH1*, most IMPCs showed 4+ (6/15, 40%) (Fig 3A); the second more frequent results were 3+ and 2+ with 3 cases each (20%); while 1+ and 0+ were observed in 2 (13.3%) and 1 (6.7%) cases, respectively. Regarding the *ZEB2* staining, most of canine IMPCs showed 0+ (11/15, 73.3%); three (20%) and one (6.7%) cases showed 1+ and 2+, respectively (Fig 3B). The immunohistochemistry and ISH results for E-cadherin and ZEB2 are showed in Table 4. Statistical correlations between *CDH1* and *ZEB2*-mRNA expressions were not observed *(p = 0*.*79; r = 0*.*07)*. Immunohistochemical and ISH results did not show a statistical correlation with *CDH1* and *ZEB2 (p = 0*.*47; r = -0*.*20)*.

**Table 4:** Immunohistochemical (IHC) and RNA *In Situ* Hybridization (ISH) staining for ZEB2 and E-cadherin (*CDH1*) in invasive areas of IMPC of the canine mammary gland.

Case	IHC Scores		ISH Scores***	
	ZEB2^[^^21^^,^^35^^]^*	E-cadherin ^[^^34^^]^**	*ZEB2*	*CDH1*
**Case 1**	+	1+	0	1+
**Case 2**	+	2+	1+	2+
**Case 3**	+	1+	0	3+
**Case 6**	+	1+	0	4+
**Case 7**	+	1+	0	3+
**Case 8**	+	1+	0	0
**Case 10**	-	1+	2+	2+
**Case 11**	-	1+	1+	4+
**Case 12**	-	1+	0	3+
**Case 13**	-	2+	0	4+
**Case 14**	-	1+	0	4+
**Case 15**	-	1+	0	2+
**Case 17**	-	3+	0	1+
**Case 18**	-	3+	1+	4+
**Case 19**	-	1+	0	4+

*(+) Presence of nuclear labelling; (-) absence of nuclear labelling.

** (0) no detectable labelling; (+1) detectable labelling in ≤10% of the neoplastic cells; (+2) detectable labelling in 10–50% of the neoplastic cells; (+3) detectable labelling in >50% of the tumour cells.

*** (0) No staining or less than 1 dot to every 10 cells (60X magnification); (1+) 1–3 dots/cell (visible at 20–60X magnification); (2+) 4–10 dots/cell and very few dot clusters (visible at 20–60X magnification), (3+) >10 dots/cell and less than 10% positive cells have dot clusters (visible at 20X magnification); and (4+) >10 dots/cell and more than 10% positive cells have dot clusters (visible at 20X magnification).

### Relationship among gene expression (*ZEB2* and *CDH1)* and clinicopathological findings of canine mammary IMPCs

As concerns immunohistochemical results a negative correlation was observed between the percentage of RP staining and E-cadherin expression (p = 0.02; r = **-** 0.53). E-cadherin ISH results showed negative correlation with animal age *(p = 0*.*006; r = - 0*.*67)*.

## Discussion

In human species, IMPCs of the mammary gland are aggressive neoplasms with high rates of lymph node metastasis and poor overall survival [25,26]. Here, canine mammary IMPCs also revealed clinicopathological characteristics related to aggressive behavior, such as lymph node metastasis and poor overall survival. These findings have been previously reported for our group in mammary IMPCs of the bitches [27,29]. Thus, it could be postulated that canine species might be a good spontaneous model to better understand the aggressive biological behavior of human breast IMPCs.

In canine mammary IMPC model our group has previously investigated, through immunohistochemistry, E-cadherin and ZEB2 protein expression [28,31]; here, we applied immunofluorescence and RNA *in situ* hybridization method to better knowing the relationship between the *CDH1* downregulation and transcriptional repression mediated by the transcription factor ZEB2. The immunofluorescence permits to compare *ZEB2* and *CDH1* expression in the same neoplastic cell; and the RNA *in situ* hybridization complement and reinforce the protein expression results obtained through immunohistochemistry.

In woman, IMPCs of the breast have been predominantly associated with high positivity for E-cadherin [42–46]. Differently, our research revealed that canine mammary IMPCs appear to be associated with a decrease of E-cadherin protein expression (most of IMPCs revealed ≤10% E-cadherin-positive neoplastic cells); ISH method revealed high rates of mRNA transcripts of *CDH1*. We postulate that these differences could be associated with the inhibition of mRNA expression that might be performed by translational repression mediated by microRNAs [47].

The transcription factor ZEB2 has been predicted to be a transcriptional repressor of the *CDH1* in human glioblastomas, breast cancer and renal cell carcinomas [48–50]. Here in order to evaluate its activity in canine mammary IMPCs, an immunofluorescence analysis was performed, and E-cadherin^+^ZEB2^—^ and E-cadherin^+^ZEB2^+^-neoplastic cells were more frequently diagnosed. The predominance of E-cadherin^+^ZEB2^—^neoplastic cells indicated the maintenance of E-cadherin expression without ZEB2 transcriptional repression. To better understand the other result, the E-cadherin^+^ZEB2^+^ group was dichotomized based on the E-cadherin staining intensity and tendency towards a predominance of ZEB2^+^E-cadherin^weak^-neoplastic cells was observed, indicating that, when present, ZEB2 expression usually induces E-cadherin downregulation in canine mammary IMPCs.

The ZEB2 is one of the transcription factors associated with transcriptional repression of *CDH1* in neoplastic diseases [51]. In human species, nuclear expression of this molecule has been reported in squamous cell carcinomas, ovarian cancer, pancreatic cancer, renal cell carcinomas and bladder cancer [52–56]. To date, in IMPCs of the canine species the expression of *ZEB2* was investigated only through immunohistochemistry being reported nuclear and cytoplasmic staining [28,31]. Here immunohistochemically 47% of the canine mammary IMPCs showed nuclear positivity for the protein ZEB2; as concerns *ZEB2* gene, mRNA transcripts were rarely observed. We postulate that the heterogeneity between the expressions of these molecules might be explained by the rapid degradation of the mRNA after its conversion in protein.

Here a negative correlation between E-cadherin protein expression and PR was demonstrated. Differently, in human breast cancer E-cadherin loss has been associated with tumor undifferentiation and negativity for hormone receptors [3]. According to our previous studies, canine mammary IMPCs are characteristically positive for hormone receptors [27]. Insulin-like growth factor I (IGF-IR) receptor expression has been associated with maintaining of E-cadherin expression and loss of hormonal receptors in breast cancer [57]. In canine mammary IMPCs we postulate that the decrease of IGF-IR expression could be related to both PR expression and E-cadherin loss. To confirm this possibility, it would be necessary to investigate the IGF-IR expression in canine mammary IMPCs.

## Conclusions

Based on our findings, the canine model of the mammary IMPCs is an aggressive neoplasm that is frequently associated with lymph node metastasis and poor overall survival. This neoplasm appears to show E-cadherin lost and, at times reveals nuclear positivity for the transcription factor ZEB2 that seems to exert transcriptional repression in *CDH1*. To better understand the transcriptional repression event and its relation to the IMPC aggressive biological behavior, more investigations should be performed in human and canine mammary IMPCs. Canine mammary IMPCs positive for PR tends to show E-cadherin loss.
