# Correction: The investigation of transcriptional repression mediated by ZEB2 in canine invasive micropapillary carcinoma in mammary gland

**DOI:** 10.1371/journal.pone.0214567

**Published:** 2019-03-25

**Authors:** Conrado de Oliveira Gamba, Karine Araújo Damasceno, Izabel Cristina Ferreira, Michele Angela Rodrigues, Dawidson Assis Gomes, Mariana Resende Alves, Rafael Malagoli Rocha, Alessandra Estrela Lima, Enio Ferreira, Geovanni Dantas Cassali

The affiliation for the seventh author is incorrect. The correct affiliation for Rafael Malagoli Rocha is: Federal University of São Paulo–Gynaecology Department, Botucatu, São Paulo, Brazil.
